# Gender equity in sport from the perspective of European women athletes and sport managers, physical education teachers and sport coaches

**DOI:** 10.3389/fpsyg.2024.1419578

**Published:** 2024-08-09

**Authors:** Raquel Vaquero-Cristóbal, Adrián Mateo-Orcajada, Yeter Aytül Dağlı Ekmekçi̇, Antonino Pereira, Shirin Amin, Lourdes Meroño, Noelia González-Gálvez, Özgür Mülazımoğlu Ballı, Francisco Mendes, Orlando Mbah, Lucía Abenza-Cano, Alejandro Leiva-Arcas, Yeliz İlgar Doğan, Abel Figueiredo, Cristina María Ponce-Ramírez, Francisco Esparza-Ros, Mario Albaladejo-Saura

**Affiliations:** ^1^Research Group Movement Sciences and Sport (MS&SPwORT), Department of Physical Activity and Sport, Faculty of Sport Sciences, University of Murcia, Murcia, Spain; ^2^Facultad de Deporte, UCAM Universidad Católica de Murcia, Murcia, Spain; ^3^Faculty of Sport Sciences, Pamukkale University, Pamukkale, Türkiye; ^4^Polytechnic Institute of Viseu, Viseu, Portugal; ^5^Champion Factory Ireland, Dublin, Ireland

**Keywords:** sports, gender, equity, European, women, athletes

## Abstract

**Introduction:**

For years, gender inequality has conditioned women’s participation in sport, exposing them to difficult situations and numerous barriers to face. However, no previous research has analyzed the situation of women in sport from the perspective of the athletes themselves, or from the perspective of the coaches, teachers or managers who work with them.

**Methods:**

This study examines the perspectives of European women athletes, sports managers, physical education teachers, and sports coaches, on gender equity in sports across six European countries: Greece, Ireland, Italy, Portugal, Spain, and Turkey. The research design was consistent with a critical realist epistemology, and the sampling method was non-probabilistic by convenience. A total of 42 female athletes (mean age: 24.37 ± 8.27 years old; mean sport experience: 6.67 ± 7.76 years) and 45 sports managers, physical education teachers or coaches (mean age: 47.00 ± 11.99 years old; mean sport experience: 9.62 ± 10.60 years), participated in six focus groups in the same countries, in groups of 6 to 10 participants per focus group. Focus groups were conducted to stimulate collective discussions, build upon and question ideas, and reach a consensus on questions drawn up by a group of experts, following previous methodologies. The data analysis involved transcribing, translating, and contextualizing the focus group recordings into English. Inductive thematic analysis, reflexive thematic analysis, and codes and themes within the data were created using NVivo 12 Pro.

**Results:**

The main topics discussed by the female athletes were “gender inequality in general and in sport,” “barriers to gender equity,” “reasons for abandonment,” “needs,” “environment role models” and “tools for the gender equity in sport.” The main topics discussed by the sports managers, physical education teachers, and coaches were “gender inequality in general,” “gender inequality in sports,” “tools” and “reasons, motives, drivers.” The results of the study revealed that gender inequality in sports is influenced by a broader social context, where stereotypes, biases, and discrimination persist. The participants also highlighted the challenges, barriers, and needs that women athletes face in their careers, such as a lack of resources, support, visibility, and recognition. Moreover, the participants suggested some strategies to promote gender equity in sports, such as increasing investment, awareness, and education, creating policies and legislation, fostering women’s leadership and role modeling, and developing mixed and inclusive sports projects.

**Discussion:**

According to athletes and sports managers/teachers/coaches, gender inequality is still present in sport. There are barriers and challenges that need to be addressed such as lack of resources, visibility, and recognition for female athletes. Among the strategies that can be used to reverse this situation are increasing investment, awareness, and education, creating policies and legislation, fostering women’s leadership, and developing mixed and inclusive sport projects.

## 1 Introduction

Sport has traditionally been a sector dominated by men, and progress in gender equality in this area is hampered by the existence of social constructs that often associate sport with the “masculine,” related to characteristics such as strength, endurance, and speed, which occur in highly competitive and confrontational contexts (Fink, 2008; Amin et al., 2023). This has been attributed to the sociocultural origin of modern sport, in which activities are considered to be more masculine if they are involving strength, endurance, and physical contact, while those involving esthetics, concentration, and flexibility are viewed as feminine (Plaza et al., 2017). This relationship also has a clear cultural bias, since it has been observed that although there is a general tendency to classify sports as masculine or feminine depending on the predominant characteristics, the fact that they are practiced mostly by men or women in certain geographical areas also affects the perception of both men and women in this respect (Matteo, 1988). Also, historically, the socialization framework related to sport, to which values such as success or prominence are attributed, has been eminently androcentric, contributing to the different participants in educational-sports environments perpetuating and justifying stereotypes from an early age (Díez-Mintegui, 2003). In addition, it has also been observed that the tendency to make strict classifications regarding what are masculine sports and what are not, is more present in men who practice them, while women do not find these limitations so evident, being this fact what some authors call implicit stereotypes (Koivula, 1995; Hardin and Greer, 2009). Also, the representation on the media often portrays sports in a way that reinforces these stereotypes, focusing on male athletes and sports that emphasize physical prowess, perpetuating the association between sports and masculinity (Koivula, 1999).

The consequences of this reality are negative for everyone (Amin et al., 2023). Firstly, for women, due to the impossibility of enjoying the same conditions and benefits as men (namely social, economic, and cultural), due to their involvement in sport and the free expression of their potential in multiple dimensions (athletes, referees, coaches, managers; Amin et al., 2023). In this regard, differences have been observed in the participation of men and women in sports from an early age. For example, in a study performed with 632 adolescents (317 boys and 315 girls), it was observed that the sports most practiced by the boys were football (24.3% boys, 1.6% girls), followed by basketball (12.0% boys, 2.5% girls) and martial arts (8.5% boys, 4.7% girls), while the most practiced sports by girls were rhythmic and expressive activities (18.0% girls, 2.5% boys), music supported activities (17.6% girls, 0.9% boys), and hiking (10.1% girls, 4.8% boys; Mateo-Orcajada et al., 2021a). Several studies have highlighted the gender stereotypes of adolescents as one of the most influential factors when selecting the sport modality to practice (Plaza et al., 2017). Gender stereotypes are commonly defined as the set of behaviors or attitudes developed and attributable to male or female individuals, framed in a given social context, which has been used to classify them, and being on numerous occasions the origin of differences between men and women (Herdt, 1996). The gender stereotypes are integrated from childhood, and result in the conception that there are male and female sports (Mateo-Orcajada et al., 2021b). Previous research have highlighted that the environment closest to the adolescents, consisting of fathers, mothers, families, siblings, friends, and teachers/coaches and the media, appear to be related to gender stereotypes and its transmission (Mateo-Orcajada et al., 2021a,b). In this regard, women teachers and coaches perceived the presence of gender stereotypes in the sport practice, hindering the possibilities of development of the girls, while the men coaches and teachers manifested the believe that there are differences in the possibilities of physical development of boys and girls, what could negatively affect to the way they teach sport (Mateo-Orcajada et al., 2021b). On the other hand, previous studies also analyzed the influence of gender stereotypes and parents’ level of sports practice on their children’s sports practice (Mateo-Orcajada et al., 2021a). The study shows that mothers’ gender stereotypes influence adolescents’ levels of sports practice, but not their gender stereotypes, being the girls more influenced than boys by their parents’ stereotypes. This could be attributed to the fact that the awareness-raising proposals on equality in sports that have been develop in recent years are leading to changes in the perception of gender stereotypes in sports (Balish et al., 2016).

Then, for society, because an inexorable human purpose is not fulfilled, that of creating conditions of justice in access opportunities (Amin et al., 2023), what may be affecting other areas of well-being, such as psychological health. Even though women’s participation in sport has gradually increased in recent years, they continue to be underrepresented in the decision-making bodies of sporting institutions at local, national, and international levels (Betzer-Tayar et al., 2017).

Along the same line, as a profession, sports coaching is dominated by men. Based on previous studies, it is estimated that only around 35% of all sports head coaches are women (Betzer-Tayar et al., 2017), and that female coaches are more likely to be found in sports that have a high proportion of female participants and in individual sports (e.g., dance, gymnastics, and figure skating; Reade et al., 2009). Something similar has been observed in governance and management positions in international federations (IF). The number of women in the top governance of IF ranges from 1 (Rugby and Wrestling) to 10 and an average of less than 5; the minimum percentage value of women in top governance of IF’s is 9% (Rugby), and the average is still 27% of the top governance of the representative sports in the Summer Olympic Games, rarely achieving 50% (Rowing) while only 2 IFs (Golf and Triathlon) have a woman as President (Pereira et al., 2023).

Obstacles to full equality are present across many sports. The problems and issues identified in sporting practice reflect the challenges we face as a society and the impact of stereotypes and social gender roles (Meier, 2005). Transforming our World: The 2030 Agenda for Sustainable Development (United Nations, 2015), establishes as a priority the promotion of gender equality, the abolition of gender stereotypes, and non-discrimination based on orientation sexuality, gender identity and expression and sexual characteristics. At the community level, the Gender Equality Strategy 2020–2025 (European Comission, 2020) reinforces the importance of the participation of women and girls in sport, as well as the balance between men and women in leadership positions within sports organizations. However, the result of this policies remains unclear (Bekiari, 2023), highlighting the need for more research in terms of gender equity and equality in the different fields related to sport.

It is therefore necessary to promote more studies that aim to understand this highly complex phenomenon and that help to foster a greater participation and involvement of women in sports, as athletes, coaches, referees, or judges, and leadership roles in sports organizations. However, to the knowledge of the authors, no research studies have included sport professionals from the different sectors, such as athletes at different levels, physical education teachers, coaches, and managers.

Thus, the main objective of this research was to discover the perception of male and female coaches, teachers, and managers, as well as women athletes, on the inequality in sports according to their experiences, and to identify the needs and tools necessary to reduce the gender gap that they could implement. More specifically, the aims were to (i) understand the opinion of sports agents from various European countries regarding gender inequality in sport; (ii) identify the challenges, obstacles, and needs of female athletes in their careers; (iii) learn about examples of good practices in the field of gender equality in sport; (iv) discover strategies and suggestions to support gender equality in sport. To give an answer to the aforementioned objectives, the research questions were (i) what may be the opinion of women athletes, coaches, trainers, physical education teachers and managers about gender inequality in sport? (ii) Which are the challenges, obstacles and needs that they think women athletes have to face? (iii) What are the practices that they implement/would implement in the field of gender equality in sport? (iv) What are the strategies that, from their experience, may support gender equality in sport?

## 2 Materials and methods

### 2.1 Design

The current study employed a cross-sectional design. The sampling was non-probabilistic by convenience, with the participation of women athletes and ex-athletes and sports managers/physical education teachers/coaches who could be accessed. The institutional ethics committee of the lead institution of this project (Catholic University of Murcia) approved the research design according to the World Medical Association and the guidelines of the Helsinki Declaration (code: CE022317).

Before starting the research process, all participants were informed of the procedure to be followed, all questions were answered, and they all signed an informed consent to participate in this research. In the case of minors, the informed consent was signed by both the minor and his/her parents. The participants were free to opt out at any time without providing any reason. The rights and welfare of the research participants were protected at all times, and confidentiality was ensured and maintained throughout the research study.

### 2.2 Participants

The inclusion criteria for the sportswomen were: (a) female athletes or ex-athletes, (b) amateur or elite; and (c) from an individual or team sport. Women athletes were excluded if they resided outside of the Europe, or if their association with or experience of women’s athletes was outside Europe.

Regarding the group of sports managers, physical education teachers, and coaches, the inclusion criteria were (a) be experts working with women in sport, either in women’s or mixed classes/teams/federations. An expert was defined as a person who had information and knowledge in a substantive area beyond that of the average person and who regularly shared this information and knowledge through consultation, teaching, or public speaking, or publications and written reports (Capranica et al., 2022). The exclusion criteria for this group were: (a) working exclusively with male athletes; (b) residing outside Europe or professional experience outside Europe.

With regard to the selection of the participants, following the methodology from previous studies (Forsyth et al., 2023), the participants were recruited via social and mass-media channels and by direct emails in six different countries: Greece, Ireland, Italy, Portugal, Spain, and Turkey. More specifically, announcements were placed on the social networks of the participating institutions and/or research groups and emails were sent to databases of the associations and universities participating in this study to indicate that volunteers were requested for the development of debates made up of female athletes; and sport managers, physical education teachers and sport coaches, indicating the inclusion and exclusion criteria, as well as the contact email in case they wished to participate.

Once the list of people willing to participate per country was available, and to ensure the proper representation of participants, a purposeful sampling technique was deemed appropriate and a core strength for gaining comprehensive, meaningful, and practical knowledge (Parker and Tritter, 2006). Thus, a selection of people for the focus groups was made based on the heterogeneity of profiles, following the methodology from previous studies (Capranica et al., 2022).

In the present work, the participants’ opinion was about the external topic, not including demographic queries about income or other personal information. Furthermore, according with previous studies, this methodology allows describing the participants’ subjective viewpoints and experiences, such as their intentions, hopes, concerns, feelings, and beliefs (Capranica et al., 2022).

Finally, a total of 42 female athletes (mean age: 24.37 ± 8.27 years old; mean sport experience: 6.67 ± 7.76 years) participated in the six focus groups (one per country), conducted with athletes in Greece, Ireland, Italy, Portugal, Spain, and Turkey, with 6 to 8 athletes per focus group. Of these, 36 were active athletes while 6 were ex-athletes; 26 were or had been involved in individual sports and 16 in team sports; and 13 were amateur athletes, while 29 were elite athletes.

In addition, 45 sports managers, physical education teachers, or coaches (mean age: 47.00 ± 11.99 years old; mean sport experience: 9.62 ± 10.60 years) participated in six other focus groups in the same countries, with 6 to 10 participants per focus group. Of these, 20 were sports managers; 15 were physical education teachers, and 10 were coaches. In total, 22 men and 23 women participated in these focus groups.

### 2.3 Instruments

In exploring the experiences of women athletes and sport managers, physical education teachers, and sport coaches, the research design was consistent with a critical realist epistemology, which allows gaining some insights into what is occurring and a good understanding of a context from the personal experiences and perspectives of individuals knowledgeable in the area under investigation (Sayer, 2000; Maxwell, 2013). More specifically, the present research was based on a focus group with female athletes and a focus group with sport managers, physical education teachers, and sport coaches, in six different European countries, namely Greece, Ireland, Italy, Portugal, Spain, and Turkey.

Considering that a large number and broad composition of focus groups increases the representativeness and validity of the findings, focus groups composed of 6–10 participants were deemed appropriate to ensure the greatest amount of information gathered from group dynamics, universal participation, and positive interaction between participants (Capranica et al., 2022).

The assumptions that framed this research mainly followed an eminence-based approach that provided insight into five main themes: (1) gender inequality in sport; (2) gender inequality in society; (3) challenges/barriers/needs in the careers of women athletes; (4) examples of good practice in gender equality in sport; and (5) strategies/suggestions to support sports activities and gender equality.

Prior to conducting the focus groups, and based on the previously specified topics, a group of experts with previous experience in conducting focus groups designed the questions to guide the discussion of the athlete focus groups, and sport managers, physical education teachers and sport coaches. The expert group consisted of 30 researchers with previous experience in designing and conducting focus groups from the six countries participating in this study. For this purpose, a face-to-face meeting was held with all the experts to agree on the questions to be asked in both types of focus groups. This meeting lasted 4 h. A draft set of questions emerged from this meeting, which was discussed again in a second online meeting with the same group of experts 4 weeks later. This meeting resulted in the final version of the questions that were used in the focus groups of athletes and the focus groups of sport managers, physical education teachers and sport coaches.

The following questions to guide the discussion of the athlete focus groups: (1) Do you think there is gender inequality in sports? If yes, do you think this situation reflects the structure in society? Why?; (2) What are the reasons that lead women to quit from their career as an athlete? Challenges that women athletes face during their career; (3) What barriers must women athletes face?; (4) What are the needs that women athletes have?; (5) Do you believe that society has helped/hindered you in any way? Please share your opinion; (6) Is there a role model/person that positively or negatively influenced you in your sport practice?; and (7) Would you like to share with us some strategies/suggestions to support sports activities and gender equality through sports?. The questions that were designed for the focus group of sport managers, physical education teachers and sport coaches were: (1) Do you think there is gender inequality in sports? If yes, do you think this situation reflects the structure in society? Why?; (2) Do you think there is enough awareness of gender inequality in sport?; (3) Why do you think gender equality in sport should be addressed?; (4) What would be very useful to promote gender equality in sport?; (5) Do you think that some seminars or informative talks on this topic would help more experts become aware of gender equality, and how could women be included in sport? What else can be done?; and (6) In your club/team/federation/education center, how is gender equality/equity promoted or otherwise not facilitated?

The questions were developed not just to document the participants’ experiences in the subjective sense, but also to explain life trajectories (Wiltshire and Ronkainen, 2021). The questions followed a semi-structured approach which included the main questions, flexible probing questions, and clarification questions, following previous examples (Didymus, 2017). This semi-structured approach allowed interviewees to discuss areas of perceived importance (Sparkes and Smith, 2014).

### 2.4 Procedure

The focus groups with women athletes, and sport managers, physical education teachers, and sport coaches, were conducted to stimulate collective discussions for building upon and questioning ideas, and to reach a desired consensus on a series of questions drawn up *ad hoc* by a group of experts, following previous methodologies (Capranica et al., 2022).

According to the previous studies (Tracy, 2010; Smith and McGannon, 2018), this research was guaranteed by means of: (1) external approval of the European committee; (2) the worthy topic based on equity of gender in sport; (3) coherence based on the recruitment procedures of participants, the standard operating procedures and the instructions to be provided to the participants during the focus groups, and the data collection and synthesis; (4) the sincerity between the members of the research team when designing the questions, when guiding focus groups without interfering with the participants’ opinions, and when analyzing data without a pre-established attitude; (5) the credibility in fostering different perspectives to be mirrored in the outcomes of the focus groups; (6) the resonance grounded on the involvement of women athletes with different characteristics, which allowed for a heterogeneous representation of the phenomenon; and sport managers, physical education teachers, and sport coaches, in order to have a combined view of the issue; (7) a significant contribution of the findings as precious insights for developing equity of gender in sport; and (8) the observation of fundamental ethical principles of the study regarding the benefit, fairness, and awareness, and anonymity of the participants involved in the study with encrypted information.

At the beginning of the face-to-face focus groups and following the pattern of previous research (Capranica et al., 2022), the organizers provided a 5 min presentation on the Women in Sport: Gender Perspectives and Future Expectations (WOMEN-UP) project, ERASMUS + project, within which these focus groups were carried out. The main objectives of the project, the participating entities, and the researchers who were going to be present in the focus groups were presented. After this, the participants introduced themselves briefly, so that all the participants could get to know each other and encourage interaction between them. To this end, the sportswomen were asked to tell their colleagues their name; age; sport they practiced; competitive level, and sporting career; years of experience; and whether they combined the practice of sport with some other issues, such as studies or work. In the case of the sport managers, physical education teachers, and sport coaches focus group, they were asked to tell the other group members their name; age; position they held and sport they played; processional experience; what kind of sportsmen and women they worked with; if they were previously sportsmen and women; what they had studied, and other types of training.

After that, the participants were provided with the seven questions in the case of focus groups with women athletes, and six questions in the case of the focus group with sport managers, physical education teachers, and sport coaches, which were created *ad hoc* to be addressed in an ordered sequence. Participants were presented each time an open-ended question was asked and were ensured freedom to interact directly, by sharing personal experiences and personal anecdotes, questioning one another, building upon one another’s views, agreeing, or disagreeing with opinions. No participant was obliged to take part in the discussion of any of the questions, nor was there a pre-established order of intervention, but each participant could speak when he or she wanted to. However, the moderator ensured as balanced a participation as possible among the participants by giving the floor in case several people wanted to speak at the same time. This was done following the methodology of previous research (Capranica et al., 2022). The participants’ contribution was provided in an open-ended and non-judgmental fashion, according to the operating procedures of the focus group. Thus, when all the questions had been addressed, a general discussion took place with all the participants, for them to contribute their remaining opinions on the above topics, which may had not yet been covered. In total, each focus group lasted between 60 and 90 min. The duration of the focus groups was standardized in order to avoid the interference of this factor in the results obtained (Greenbaum, 1998).

The focus groups were conducted in the country’s official language. All focus groups were conducted in isolated, open-plan rooms, where participants could sit in a circle, favoring interaction, with adequate lighting and sound. They remained seated in a calm environment, without noise or distractions that could influence their answers (Greenbaum, 1998). In all the sessions, a researcher acted as a facilitator, following the principles of neutrality and non-influence (Tracy, 2010; Smith and McGannon, 2018), without adopting a strict or relaxed approach so as not to interfere with the results (Greenbaum, 1998). In addition, the entire sessions were recorded with a password-encrypted digital recording device to facilitate the subsequent analysis of the focus groups.

### 2.5 Data analysis

To analyze the focus group data derived from the multiple focus groups carried out on the same general topic in different countries, and to maintain consistency with the original purpose of the research, for each country, two bilingual members of the research team independently transcribed, translated, and contextualized into English, in terms of the cultural aspects of the country where it was carried out, any statement (e.g., word, short phrase, or sentence) recorded during the focus groups. Afterwards, they came into an agreement on a combined version (Capranica et al., 2022).

An inductive thematic analysis was deemed necessary for organizing and interpreting the recorded statements into content units (Stewart and Shamdasani, 2014; Braun and Clarke, 2019). Thus, a reflexive thematic analysis (Braun and Clarke, 2019) was used to create codes and themes within the data using NVivo 12 Pro, which were reconstructed and revisited. Two researchers deeply immersed themselves in the data independently. More specifically, the two researchers independently read the transcripts of all the focus groups. They then conducted multiple coding sweeps, first to code surface meaning and then latent meaning. The two researchers then independently generated themes based on the previously established codes. Once they had completed this work separately, they together revised and refreshed codes and themes, and questioning each other’s assumptions and positions (Smith and McGannon, 2018). A third reviewer was consulted to resolve any disagreements. To determine the inter-rater reliability of the reviewers, Cohen’s Kappa (McHugh, 2012) was calculated, which showed a strong level of agreement (Kappa = 0.876). Finally, they coded all the texts of the focus group translations according to codes and themes created. Themes were actively developed through patterns of shared meaning, and were inductively derived, rather than being developed based on the topic guide. Anonymized quotes were extracted from the data to support the themes.

## 3 Results

The study’s data showed that athletes and sport managers, physical education teachers and sport coaches had common thoughts and statements on gender inequality, challenges, obstacles, and strategies (Tables 1, 2). In general, gender inequality was detected in all contexts, such as, work, school, education, and politics. Gender stereotypes also were detected, and it was found that there is a lack of awareness in society on this issue, which are also reflected in sports. This could create some problems, such as the number of women in management and technical fields is insufficient, and there is unequal access to financial support, psychological support, politic support, visibility in the media, environmental support, dual careers and adaptations in their training to their physiological characteristics.

**Table 1:** Themes, groups, and codes identified in the women athletes focus groups.

Theme	Group	Code	Spain	Portugal	Italy	Greece	Turkey	Ireland	Total	Cite
Society	Gender inequality in general	Influence of gender inequality in society on sport	1	2	2	0	4	9	18	“There is gender inequality in sports. Women are often paid less and have fewer opportunities for sponsorship compared to men. I think this reflects the broader gender inequalities in our society, where women are still not given equal opportunities and recognition as men” P1, Ireland
		Lack of awareness and presence of gender stereotypes leading to gender biases	2	2	0	0	5	0	9	“I always felt that I was judged more as a woman. For example, social media judged me a lot based on my body. You know, expressions like Roberto Carlos’s legs. My male gymnast friends are not considered so much. For a woman, there is actually a perspective that everyone has to fit into those patterns. So, of course, I tried very hard to break these stereotypes.” P3, Turkey
	Gender inequality in sports	Lack of awareness and presence of gender stereotypes leading to gender biases	5	2	5	1	16	2	31	“Most of our family, our grandfathers and grandmothers, think you are a woman, a girl. You know, it is expected to be successful in terms of education. “Let her become a doctor, a teacher. Let her study; let her get somewhere good.” Because this is also a financial aspect, they say, “What will sports bring you? You should have a good profession in your life.” P1, Turkey
		Lack of women in technical and senior management positions (management and training)	5	0	1	0	0	1	7	“And in relation to coaches, I think there are a lot more male coaches in my sport, but a lot because I rarely see a female coach” P2, Spain
		Gender gap in the professionalization of sport	2	0	3	0	4	0	9	“… and many times lack of respect to our work as professional players, and finally, somehow related to the first two issues, the fact that I could have never had a decent and dignified life with the (non-existing) contract the teams were offering” P6, Italy
		Priority accessibility to human and material resources and competitive environment	1	2	1	2	4	5	15	“Women also face barriers in terms of access to resources and facilities. For example, there are still many countries where it’s not safe for women to participate in sports, and female athletes may not have the same level of support and funding as their male counterparts” P2, Ireland
		Transformative gender approach in sport	12	9	0	2	10	10	43	“I agree with her, right now it is the stereotype bias, that we are always saying, boys’ sports with girls’ sports…. Although I think that nowadays this is has gone… That it is being lost because it is changing both at school and… It is no longer said: “this is for boys, this is for girls.” But even so, there are still many barriers.” P3, Spain.
		Differences in the economic sphere	4	5	0	0	2	6	17	“Of course, with the money, we got that. Money is not just a sponsorship. The money helps us to get this psychological follow-up, to have a physiotherapist, to buy the best and most suitable bike. Money, unfortunately, is quite important.” P3, Portugal
		Media visibility	3	4	0	0	0	3	10	“I think the biggest need is to have gender equality and have more opportunities for women to train and compete and more visibility.” P4, Portugal
	Barriers to gender equity	Lack of gender focus sensitive to physiological differences	2	0	3	1	3	0	9	“You know, a girl’s body gets fat after her period, a natural, normal process. But because there is a male-dominated understanding of coaching, and I think our sports sciences are mainly based on men and new research is being done on women, many people say a lot about our fat ratio” P2, Turkey
		Lack of support from close environment	2	0	1	1	3	0	7	“But my grandparents were like, “What will she do as an athlete? Let her study.” P8, Turkey
		Lack of support from technical staff and teachers	3	0	0	0	2	0	5	“But it is true that, negatively, some teachers have been surprisingly negative. They never said it to me in those words, but they did not like that I dedicated myself to swimming because they said I wasted a lot of time, that it did not let me study, that I was too tired to go to class.” P6, Spain
		Lack of availability of material resources	0	2	0	0	1	3	6	“In my sport, I have seen women with problems, especially with the federation. For example, the federation takes four boys and two girls. Because they say, boys will be more successful. Even though we have a quota, they do not give us that quota because men would be more successful.” P7, Turkey
		Lack of visibility in mass media and relevant models/referents	1	1	0	0	0	10	12	“I think one of the biggest challenges for women athletes is the lack of media coverage and recognition. Even when female athletes achieve the same level of success as male athletes, they often receive less media attention and are not given the same level of recognition. This can be demotivating and can make it difficult to sustain a long-term career in sports.” P6, Ireland
		Lack of adaptation to physiological differences	1	1	1	0	2	1	6	“I did have in the “Blume,” my physical trainer, who asked us, he asked us when our period was due, when it was over… As if to keep track of the Menstrual Cycle and then he explained to me that there were studies in which, in “x” phase, each of the phases were good for a type of work…
										But I tell you, he has been the only person who has asked me about that and has taken that into account. In other words, in the rest of the swimming world, and I have had several coaches, nobody has ever worried about that.” P6, Spain
		Lack of consideration of menstruation and motherhood	3	0	1	3	7	0	14	“Also, the fact that many of us want to start a family and face problems reintegrating after such a decision. Even coaches do not know how to handle such situations when dealing with women” P6, Greece
		Lack of research on sport physiology and women	3	0	0	0	2	0	5	“You know, a girl’s body gets fat after her period, a natural, normal process. But because there is a male-dominated understanding of coaching, and I think our sports sciences are mainly based on men and new research is being done on women, many people say a lot about our fat ratio” P2, Turkey
		Lack of awareness and presence of gender stereotypes leading to gender biases	2	0	0	1	1	7	11	“My family worried my body would deteriorate when I first started playing soccer” P5, Turkey
		Presence of gender violence and harassment	0	2	0	1	10	1	14	“For example, I ride my bicycle; I cannot go out alone when it gets dark because car drivers can tease you when they find out you are a girl. By the way, my hair is short, sometimes they may think I am a boy, but usually, if you are a girl, you are teased. I was harassed with car horn honks and stuff like that. That is why I usually train with my male friends, so I do not want to train alone a lot.” P8, Turkey
		Presence of difficulties in the development of dual careers	0	3	2	0	0	0	5	“And then, as there is effectively no career prospect, the option is always to leave sport in the background. Be it the commitment, the studies, or the work, they are obviously the first option of the athletes. Therefore, everything indicates that these are the major barriers that I have identified throughout my sports career.” P6, Portugal
		Lack of financial resources	2	1	2	2	0	6	13	“As a female athlete, I have faced many barriers in my career. One of the most significant barriers I have faced is the lack of investment in women’s sports. This makes it challenging to compete at the highest levels and limits the opportunities available for women athletes.” P5, Ireland
		Lack of women in technical and senior management positions (management and training)	0	0	0	2	0	2	4	“And in relation to coaches, I think there are a lot more male coaches in my sport, but a lot because I rarely see a female coach” P2, Spain
Sport	Reasons for abandonment	Lack of financial support	1	3	1	4	0	3	12	“Τhe obstacles faced by all athletes at competitive level one and the most basic one is the financial support to be able to continue in this category” P3—Greece
		Lack of visibility in mass media	0	1	0	0	0	2	3	“As I have said before, I think one of the biggest barriers that women athletes face is the lack of media coverage and visibility. It’s frustrating to see that male athletes and their sports are often prioritized and given more attention, while women’s sports are overlooked and deemed less valuable.” P1, Ireland
		Lack of psychological support	0	1	1	1	0	1	4	“Another point is that women should be able to get psychological support, as I said at the beginning. Because many people can become pessimistic in the face of the problems they experience. It can even get to the point of quitting sports. But at this point, I think psychological support is critical in terms of making the right decisions, in terms of being guided correctly, and in terms of thinking correctly.” P1, Turkey
		Incompatibility with the dual career	2	6	1	0	0	1	10	“I will speak from my experience. I left professional volleyball because I moved to another city to study, that is, I did not have the compatibility to continue.” P5, Portugal
		Family incompatibility	0	3	0	2	0	1	6	“Also, the fact that many of us want to start a family and face problems reintegrating after such a decision. Even coaches do not know how to handle such situations when dealing with women” P6, Greece
		Excessive demands/pressure/burnout (physical and/or psychological)	1	3	0	1	2	0	7	“And another difficulty is, for example, in the case of people who train twice a day and who trained before entering university, they cannot maintain that rhythm. And they feel that the income level will go down. That is, we know that if we start to train less, we will also perform less. But it ends because we will end up becoming demotivated because we want to keep the same pace of training.” P1, Portugal
		Pregnancy/menstruation	5	0	0	1	0	0	6	“We are talking about pregnancy, but I think the same thing happens with menstruation, because we have different phases and we are not always the same and in one phase one thing suits us better than another, and there is still a great lack of knowledge.” P6, Spain
	Needs	Increased support from close environment	0	1	0	1	2	1	5	“We also need a supportive environment that is free from discrimination, sexism, and biases. Women athletes should not have to face discrimination or sexism in their workplaces, and this includes sports. Policies and practices should be put in place to ensure that everyone, regardless of gender, is treated fairly” P5, Ireland
		Increased visibility in mass media	2	4	1	0	0	3	10	“We, for example, except for the Olympic Games, never show a swimming test on television, which is sad, but it’s the truth. So, I think they could transmit. There it was, it gave such visibility and maybe some more money for swimming, which was good.” P3, Portugal
		Accessibility to material resources and competitive environment	1	1	1	0	1	1	5	“Well, I think that one thing that is important and that… that part of resources, maybe within a coaching staff, which is already getting the physical trainer, the nutritionist, I think it is also an important part and resources are not obtained, at least in futsal.” P1, Spain
		Need for women in technical bodies and senior management positions	3	1	1	1	0	1	7	“Well, especially that there are more female coaches in the coaching staff because in the end, I think we all understand each other better and, well, that there is more variety,” P6, Spain
		Support for family and personal reconciliation	0	0	2	1	0	1	4	“Importance of having family support, especially in the presence of children and non-family supports (financing and family support services; paid leave and differentiated working hours; differentiated study paths and plans and with facilitated methods) to be able to resolve needs and requirements that show up.” P7, Italy
		Financial support	0	4	1	2	0	3	10	“Of course, with the money, we got that. Money is not just sponsorship. The money helps us to get this psychological follow-up, to have a physiotherapist, to buy the best and most suitable bike. Money, unfortunately, is quite important.” P3, Portugal
		Professionalization of technical staffs	0	1	1	0	3	1	6	“I think it is very important to train women coaches, but I think it is very important to teach them consciously. I mean, there are a lot of coaches around, but because they are not trained, especially psychological training is not provided, the right athletes are not trained.” P6, Turkey
		Safe environment free of harassment and abuse	0	0	0	0	1	10	11	“Overall, we need to create a more equitable and inclusive environment for female athletes to thrive in. Addressing these needs can help level the playing field for women in sports and ensure that they have the support and resources they need to succeed.” P7, Ireland
	Environment role models	Other women athletes	1	3	3	2	7	6	22	“Even though I continued to play football professionally, I had idolized (basketball player) a lot since I was a little girl. Işıl was my lifestyle. I was interested in her; she was successful. She was the basketball team captain; she had everything to be a role model. She had a leadership quality especially.” P5, Turkey
		Family	5	0	1	3	4	0	13	“And my mother has never complained: “I have to take Participant 5 swimming.” She has given up everything she had to do because I went to train every day and she has done the same with my brother. So I think that, thanks to the effort my family has made, I have been able to get where I am now because if my family had not said: “yes, yes, I go to Participant 5 to train every day in Albacete.” Well, surely I would never have reached the level of training that I am now. So I would say my family.” P5, Spain
		Staff	2	2	0	1	1	2	8	“I was positively influenced by a coach that I had, who took me to the National Team and I wanted to follow in her footsteps and try to get as far as possible, and then models like people who play at a professional level. When I found myself at an almost professional level, it was like I saw them… They motivated me to become like them” P3, Spain
	Tools for the promotion of gender equity in sport	Investment in women sport	0	0	1	4	0	3	8	“Campaigns to promote women’s sport, funding from society in women’s sports.” P4, Greece
		Collaboration in mixed sports projects	2	2	2	3	0	0	9	“So I think that what we should encourage is that girls know what they can do and where they can do it and that they know they can do the same as boys and that they can get rid of their fears, their doubts, their embarrassment…” P6, Spain
		Promotion of women’s sports in the mass media	1	2	0	1	0	3	7	“Another strategy is to work with media outlets and sponsors to increase coverage and support for women’s sports. This can include featuring more female athletes in advertising campaigns, providing more opportunities for interviews and coverage of women’s sports events, and increasing sponsorship opportunities for female athletes. By increasing visibility and recognition for women’s sports, we can help shift cultural attitudes and perceptions about gender in sports.” P6, Ireland
		Creation of a legislative framework that defends gender equity in sports.	1	3	1	2	0	0	7	“Therefore, for me, the question here will have to be much more about the management of support to clubs so that they can also, together with the athletes themselves, provide working conditions and the development of sports practice.” P6, Portugal
		Promoting the inclusion of women in technical staff	1	0	0	1	3	4	9	“We can also work to create more opportunities for women to take on leadership roles in sports, both on and off the field. This can include providing training and resources for women to become coaches, referees, and administrators.” P6, Ireland
		Stakeholder training on gender equity and sport and examples of good practices	0	1	0	2	2	2	7	“I think it’s important to educate coaches, officials, and other stakeholders about the importance of gender equality in sports. This can include providing training on topics such as diversity and inclusion, as well as offering resources and support to help them promote these values in their respective roles.” P5, Ireland
		Support from close environment	1	0	1	0	2	0	4	“In addition to the “private and personal” availability of family members, teachers and employers, regulatory interventions are needed at all levels: regional, national, and even European.” P7, Italy

**Table 2:** Themes, groups, and codes identified in the sports managers, physical education teachers, and coaches focus groups.

Theme	Group	Code	Spain	Portugal	Italy	Greece	Turkey	Ireland	Total	Cite
Society	Gender inequality in general	Lack of awareness and presence of gender stereotypes leading to gender biases	20	3	0	2	4	1	30	Many times, on occasions, these are things that are normalized and you do not realize that there is discrimination and that this is the case, and it has happened to me with colleagues: “well, I had not realized.” It is true that you are right about that. P1, Spain.
		Influence of gender inequality in society on sport	9	6	1	5	11	1	33	Of course, there is a need. We are in need, but unfortunately, as I said, we are in a male-dominated community. This is also due to our socio-cultural structure, as we are patriarchal. P1, Turkey.
		Gender inequality at work, school, studies, politics…	2	7	0	1	4	1	15	And of course it is a reflection of social inequality. However, with everything that is going on in this country in education, I do not know how we are going to get boys to want to work in education. The best grades are given by girls at the entrance to medical degrees. One day there will have to be quotas for some medical specialties, I’m almost certain, because I have family members who are doctors and they say that there will have to be male quotas for some medical specialties. So there is always an incredible imbalance. P7, Portugal.
		Glass ceiling for women	4	1	0	1	0	4	10	One issue is that some sports still prioritize men over women in terms of funding, resources, and media coverage. P1, Ireland.
										Because we have seen that there is a trend toward male sports and female sports. From the point of view that the data speak for themselves, I think that there are barriers that limit women, such as, as we said before, the stereotypes that society has set for us. P1, Spain.
										No, it is about being on the same starting line and having the same conditions for everyone. For us, it is often the barriers of motherhood. In other words, it is not easy. P1, Spain.
		Transformative gender approach and its influence in sport	9	2	0	1	8	0	20	But I think that nowadays I do not see that inequality in terms of physical barriers, in terms of saying “this is for boys, this is for girls.” P2, Spain.
										As the socio-cultural function of women in society develops worldwide, the situation of modern women and our mothers, fathers, and grandmothers will not be the same. They have started to lead a life that is much more integrated into business life and, therefore, sports. P4, Turkey.
	Gender inequality in sports	Lack of awareness and presence of gender stereotypes leading to gender biases	21	11	7	14	11	10	74	But there are sports that are always very categorized for women and that means that sometimes there are very few boys in rhythmic gymnastics and maybe the inequality is produced in the male category because they look at it as strange because it is there, for example in rhythmic gymnastics. P3, Spain.
										“If possible, we could openly discuss and say that women have the same opportunities as men in society. But we do not. We were born to have family, and not competing in the sports. Why?” P7, Greece.
		Lack of women in technical and senior management positions (management and training)	8	1	2	0	12	2	25	Being a female coach in high positions in elite sports is difficult. After practicing sports for many years and experiencing that stress, no one wants to leave the field and become a coach. My former teammates and I do not want to be there either. P1, Turkey.
										In 2011, I founded my own club. I realized that all the managers were men and all the coaches were men. I said, “No. I can start a sports club, and then I can do things the way I want.” I tried different things. P2, Turkey.
		Lack of gender focus sensitive to physiological differences	5	1	0	0	1	1	8	However, they have a disadvantage. As my other teachers have mentioned, after working, after stepping into marriage, men can continue their sports very comfortably. They can continue their training. But unfortunately, after marriage, after that stage of life, we cannot see this in women. We are forced to make a choice, and maybe we come to a crossroads in the most productive period of our sports life. P6, Turkey.
		Gender gap in the professionalization of sport	6	6	0	0	7	7	26	Because I can give you about a hundred incidents that I have suffered from as a woman or as a coach of women, all of which have resulted in the female athlete or the female coach being at a disadvantage compared to the male athlete. P4, Turkey.
										Of course, there are differences. There are financial differences. A men’s soccer team match is aired on beIN Sports. P7, Turkey.
										There is still much work to be done. We need to focus on promoting gender equality in all sports, including providing equal opportunities and resources for all athletes, challenging gender stereotypes and biases, and creating a safe and inclusive sports environment for everyone. P6, Ireland.
		Transformative gender approach in sport	20	12	2	6	21	8	69	Athletics is a very egalitarian sport in relation to football. For example, in athletics, both girls and boys share the same facilities, the same competition schedules. There is no discrimination in this aspect. P1, Spain.
										“Future Spike” aims to raise strong women, it is an organization for girls only. Yes, raising volleyball players is a goal there. As you can appreciate, it is not easy to train many volleyball players. But by ensuring each participating athlete’s psychological and physical development, our primary objective is to raise strong women. P2, Turkey.
										Our world federation president is also a woman regarding the equality of women and men, which we face a lot. P3, Turkey.
		Media visibility	1	3	2	1	4	3	14	Where is a women’s soccer team match aired? Most of us do not even know. P7, Turkey.
										There are still many barriers that prevent women athletes from participating fully in sports, such as unequal funding and media coverage. P2, Ireland.
		Differences in participation and dropout rates	11	6	2	0	8	0	27	From the age of 6 until the age of 11 you have 50–50 or 60–40 in female–male ratio, and suddenly there is a female break there, terrible, but even sports like athletics as an individual sport or volleyball as a more feminine sport. There is a standstill.
										It is a standstill not because of inequality but because there is something we are not getting right with the girls. I do not know what it is, but at least that is what happens to me in sports schools. They are all free, there are no luxury sports. There are 16 sports schools, there is a football pitch and there is a terrible pull with the girls. But when they reach the age of 15, there is a terrible cut-off. P3, Spain.
										The number of women athletes and women coaches is the same for tennis. It is less when compared to men. P4, Turkey.
Sport	Tools	Training of stakeholders related to sport in gender equity	15	2	13	7	6	16	59	That they move outside and that it does not matter if they play a more masculine sport… These are stereotypes and I try to work with them, especially with rugby. P5, Spain.
										Training is vital. You cannot expect a woman to take a step forward to lead a federation, a sports club, let us say, if she is not given that training. P6, Spain.
										It would be useful to combine technical courses with communication courses, from the earliest years. P8, Italy.
		Promoting equity in education/early ages	9	1	3	3	4	4	24	I think that training and information is important to promote equality. Training, this is what we are doing here and now. In parents, at schools…
										Listen to this, why, how it can be done… Training will make it happen. P3, Spain.
		Economic investment for equity in sports	0	2	0	1	0	7	10	Including increasing funding and resources for women’s sports, providing equal training and development opportunities. P7, Ireland.
										I believe that providing equal opportunities for both genders in sport is essential. We need to ensure that women have access to the same resources and support as men, from training facilities to funding. P1, Ireland.
		Equality policies in sports	5	7	7	4	1	13	37	First by developing policies to reduce gender inequalities, focusing on the greater presence of women in decision-making roles to make them promoters of change, encouraging the new generations to remain in the world of sport beyond their competitive careers. P3, Italy.
										This could involve developing and enforcing clear policies and protocols, providing training and resources for coaches and officials, and involving athletes and their families in the decision-making process. P7, Ireland.
		Encourage the presence of more women in the sports field, both in training and in management and direction	3	6	2	3	2	12	28	We need women and men to drive change. From women in prominent places, decision-making positions and leadership positions to pave the way for new generations, to be able to make decisions that allow other women to have the opportunities they want and make their choices freely. P4, Portugal.
										I think we need to involve more women in decision-making and leadership positions in sports organizations and institutions. This would not only increase the representation and voice of women in sport, but also bring diverse perspectives and ideas to the table. P5, Ireland.
		Role modeling of female athletes and media attention in mass media	3	1	1	3	2	15	25	One way to promote gender equality in sport is to encourage more girls to participate in sports at a young age. This can be achieved by providing accessible and affordable programs for girls, as well as creating role models for them to look up to. P3, Ireland.
										It would be very useful to have more visible and accessible female role models in sport. Women athletes who are successful and highly respected could inspire and motivate young girls and women to participate in sport, pursue athletic careers, and challenge gender stereotype. P1, Ireland.
		Mixed teams in pre-competitive stages	9	2	1	1	1	0	14	And we play: “this school and this school” and “5th grade A plays against 5th grade B of this school,” “twenty against twenty, be they boys, be they girls, be they red, be they green, be they orange….” We have to play with this too, with the culture that exists in the villages… P3, Spain.
										At school age, for me, it is crucial that there are no boys’ sports and no girls’ sports, thus maximizing the practice of sport, always adapting it to age, not to gender. P6, Spain.
		Creating safe and inclusive environments: gender-based violence and bullying	1	1	1	0	0	8	11	I agree with all the previous suggestions, but I would also add that we need to create safe and supportive environments for girls and women in sports, free from harassment, abuse, and discrimination. P7, Ireland.
										It is important to create a supportive and inclusive environment for women athletes to encourage their full participation in sports. P4, Ireland.
	Reasons/motives/drivers	Transformative power of sport for society	3	0	3	1	1	7	15	Gender equality in sport should be addressed because it is necessary for promoting international development and cooperation. Sports have the power to bring people together from all corners of the globe, and addressing gender inequalities can help to ensure that all individuals have equal access to these opportunities. Addressing gender inequalities can also help to create a more peaceful and just world by promoting respect, understanding, and cooperation between different cultures and nations. P6, Ireland.
		Opportunities for access to sports practice	3	1	3	2	1	10	20	Gender equality in sport should be addressed because it is necessary for creating positive role models for future generations. When women are represented and successful in sports, it sends a powerful message to young girls that they too can achieve greatness in their chosen field. Addressing gender inequalities can also help to break down stereotypes and barriers that limit women’s participation in sport and other male-dominated fields. P5, Ireland.
										Promoting gender equality in sports can help to inspire new generations of female athletes and promote positive change both on and off the field. P8, Ireland.
										When we provide equal opportunities for women athletes, we tap into a huge pool of talent and potential that might otherwise go untapped. P2, Ireland.
		Eradication of gender stereotypes	3	0	0	0	0	4	7	To inform about the inequalities that exist in sport, to explain how these inequalities can be alleviated. Not to normalize things and aspects that are purely sexist. Make them see which things are sexist and which are not. P1, Spain.

### 3.1 Results for female athletes

Table 1 summarizes the results of the themes, code groups, and codes identified in the various focus groups conducted with the sportswomen. In this regard, the main themes “Society” (*n* = 270 citations) and “Sport” (*n* = 200 citations) were identified.

Through the information provided by the sportswomen, the code groups “Gender inequality in general” (*n* = 27 citations), “Gender inequality in sport” (*n* = 132 citations) and “Barriers to gender equity” (*n* = 111 citations) were identified within the “Society” theme. On the other hand, the code groups “Reasons for abandonment” (*n* = 48 citations), “Needs” (n = 58 citations), “Environment role models” (*n* = 83 citations) and “Tools for the promotion of gender equity in sport” (*n* = 51 citations) were identified within the “Sport” theme.

The codes identified through the participants’ interventions were “Influence of gender inequality in society on sport” (*n* = 18 citations) and “Lack of awareness and presence of gender stereotypes leading to gender biases” (*n* = 9 citations), in the group “Gender inequality in general.”

“Lack of awareness and presence of gender stereotypes leading to gender biases” (*n* = 31 citations), “Lack of women in technical and senior management positions (management and training)” (*n* = 7 citations), “Gender gap in the professionalization of sport” (*n* = 9 citations), “Priority accessibility to human and material resources and competitive environment” (*n* = 15 citations), “Transformative gender approach in sport” (*n* = 43 citations), “Differences in the economic sphere” (*n* = 17 citations) and “Media visibility” (*n* = 10 citations) were included in the group “Gender inequality in sports.”

“Lack of gender focus sensitive to physiological differences” (*n* = 9 citations), “Lack of support from close environment” (*n* = 7 citations), “Lack of support from technical staff and teachers” (*n* = 5 citations), “Lack of availability of material resources” (*n* = 6 citations), “Lack of visibility in mass media and relevant models/referents” (*n* = 12 citations), “Lack of adaptation to physiological differences” (*n* = 6 citations), “Lack of consideration of menstruation and motherhood” (*n* = 14 citations), “Lack of research on sport physiology and women” (*n* = 5 citations), “Lack of awareness and presence of gender stereotypes leading to gender biases” (*n* = 11 citations), “Presence of gender violence and harassment” (*n* = 14 citations), “Presence of difficulties in the development of dual careers” (*n* = 5 citations), “Lack of financial resources” (*n* = 13 citations) and “Lack of women in technical and senior management positions (management and training)” (*n* = 4 citations) were included in the group “Barriers to gender equity.”

The codes “Lack of financial support” (*n* = 12 citations), “Lack of visibility in mass media” (*n* = 3 citations), “Lack of psychological support” (n = 4 citations), “Incompatibility with the dual career” (*n* = 10 citations), “Family incompatibility” (*n* = 6 citations), “Excessive demands/pressure/burnout (physical and/or psychological)” (*n* = 7 citations) and “Pregnancy/menstruation” (*n* = 6 citations) were included in the group “Reasons for abandonment.”

“Increased support from close environment” (*n* = 5 citations), “Increased visibility in mass media” (*n* = 10 citations), “Accessibility to material resources and competitive environment” (*n* = 5 citations), “Need for women in technical bodies and senior management positions” (*n* = 7 citations), “Support for family and personal reconciliation” (*n* = 4 citations), “Financial support” (*n* = 10 citations), “Professionalization of technical staffs” (*n* = 6 citations) and “Safe environment free of harassment and abuse” (*n* = 11 citations) were included in the group “Needs.”

“Other women athletes” (*n* = 22 citations), “Family” (*n* = 13 citations) and “Staff” (*n* = 8 citations) were included in the group “Environment role models.”

“Investment in women sport” (*n* = 8 citations), “Collaboration in mixed sports projects” (*n* = 9 citations), “Promotion of women’s sports in the mass media” (*n* = 7 citations), “Creation of a legislative framework that defends gender equity in sports” (*n* = 7 citations), “Promoting the inclusion of women in technical staff” (*n* = 9 citations), “Stakeholder training in gender equity and sport and examples of good practices” (*n* = 7 citations) and “Support from close environment” (*n* = 4 citations), were included in the group “Tools for the promotion of gender equity in sport.”

### 3.2 Results for sports managers, physical education teachers, and coaches

The themes, groups, and codes identified in the sports managers, physical education teachers, and coaches’ focus groups are summarized in Table 2. The main themes were society (*n* = 351 citations) and sport (*n* = 250 citations).

Regarding the groups, “gender inequality in general” (*n* = 108 citations) and “gender inequality in sports” (*n* = 243 citations) were included in society; while “tools” (*n* = 208 citations) and “reasons, motives, drivers” (*n* = 42 citations) were included in sport.

“Gender inequality in general” was composed of the codes “lack of awareness and presence of gender stereotypes leading to gender biases” (*n* = 30 citations), “Influence of gender inequality in society on sport” (*n* = 33 citations), “Gender inequality at work, school, studies, politics…” (*n* = 15 citations), “Glass ceiling for women” (*n* = 10 citations), and “Transformative gender approach and its influence in sport” (*n* = 20 citations).

“Gender inequality in sports” was composed of the codes “Lack of awareness and presence of gender stereotypes leading to gender biases” (*n* = 74 citations), “Lack of women in technical and senior management positions (management and training)” (*n* = 25 citations), “Lack of gender focus sensitive to physiological differences” (*n* = 8 citations), “Gender gap in the professionalization of sport” (*n* = 26 citations), “Transformative gender approach in sport” (*n* = 69 citations), “Media visibility” (*n* = 14 citations) and “Differences in participation and dropout rates” (*n* = 27 citations).

“Tools” was composed of the codes “Training of stakeholders related to sport in gender equity” (*n* = 59 citations), “Promoting equity in education/early ages” (*n* = 24 citations), “Economic investment for equity in sports” (*n* = 10 citations), “Equality policies in sports” (*n* = 37 citations), “Encourage the presence of more women in the sports field, both in training and in management and direction” (*n* = 28 citations), “Role modeling of female athletes and media attention in the mass media” (*n* = 25 citations), “Mixed teams in pre-competitive stages” (*n* = 14 citations) and “Creating safe and inclusive environments: gender-based violence and bullying” (*n* = 11 citations).

“Reasons, motives, drivers” was composed of the codes “Transformative power of sport for society” (*n* = 15 citations), “Opportunities for access to sports practice” (*n* = 20 citations) and “Eradication of gender stereotypes” (*n* = 7 citations).

## 4 Discussion

The main objective of the present research was to discover the perception of male and female coaches, teachers, and managers, as well as women athletes, on the inequality in the sport field according to their experiences, and to identify the needs and tools to reduce the gender gap that they could implement. The study’s data showed that athletes and managers had common thoughts and statements on gender inequality, challenges, obstacles, and strategies. These results are similar to those of previous research in which it was observed that in the adolescent population, as well as in their teachers and coaches, gender stereotypes were still present, highlighting the male role over the female role in sport (Mateo-Orcajada et al., 2021a,b). One possible explanation for this is that, although gender equality policies exist, actions to achieve gender equality are more limited and patriarchal language, gender stereotypes and profiling of people still persist, making it difficult for women to work in this area (Evans and Pfister, 2021). Therefore, despite the fact that in recent years there have been campaigns and promotions in search of equity in sport, in the light of the results of this research, these actions have not yet achieved the desired objective, and there still more barriers for women to overcome (Boiché et al., 2014; Leiva-Arcas et al., 2021; Mateo-Orcajada et al., 2021a; Costa and Miragaia, 2022).

According to the participants’ statements, gender inequality exists in all areas, such as work, school, education, and politics. The topic of gender equity in sports is widely and globally discussed and researched in various academic fields, such as sociology, psychology, management, education, and law (Yiamouyiannis and Osborne, 2012; Burnett, 2018; Cunningham et al., 2021; Jeanes et al., 2021). Thus, this highlights the complexity and diversity of the issues related to gender equity in sport, as well as the need for interdisciplinary and collaborative approaches to address them (Yiamouyiannis and Osborne, 2012; Burnett, 2018; Cunningham et al., 2021; Jeanes et al., 2021). This social canker is not just a Euro-centric phenomenon, but a global issue that requires a holistic, unfettered, and interdisciplinary approach to address (Yiamouyiannis and Osborne, 2012; Burnett, 2018; Cunningham et al., 2021; Jeanes et al., 2021).

Furthermore, data from the present research show that gender stereotypes are also reflected in sports according to the opinion of both sportswomen and sports managers/professions/coaches. In addition, it was found that gender stereotypes exist, and there is a lack of awareness in society on this issue. When the literature is reviewed, many studies with similar results are found, which could demonstrate that gender inequality is not an issue of a specific area, but an issue that needs to be addressed in a multidimensional manner (Evans and Pfister, 2021; Jeanes et al., 2021; Graham and Blackett, 2022). Evans and Pfister's (2021) evidence in the literature that women are underrepresented in leadership positions globally and that men are aware of the problem, patriarchal selection practices and organizational cultures reinforce this inequality. One of the reasons for this may be that female athletes and coaches who grow up in a patriarchal sports environment may unwittingly accept masculine norms and may not fully grasp the dimensions of inequality (Graham and Blackett, 2022). Another reason is that, even when women retire from sport, fewer female players than male players can transfer their skills into well-paid coaching and managerial roles, even though they have achieved equivalent success as men (Harrison et al., 2022).

A relevant finding of this research was that in their opinion, the number of women in management and technical fields is insufficient, and there is unequal access to resources. Previous studies have emphasized men’s control and authority in mixed-gender sports fields, showed that women’s participation in sports in different positions (athlete, manager, or coach) is not enough to achieve gender equality. However, it is emphasized that while the number of female athletes has increased in recent years, the fact that female head coaches at national and international levels have not increased similarly is primarily due to institutional and societal reasons (Culver et al., 2019). Policies that seek to promote gender equality in sports need to focus not only on increasing women’s participation but also enforcing structural changes in sports that allow sportswomen to have references in all areas of sport (Jeanes et al., 2021).

From the participants’ responses to the question “What are the views of the participants on the challenges/obstacles/needs of women athletes in their careers?,” it was concluded that there is a lack of financial support, physiological differences are not taken into consideration, psychological support, visibility in the media, environmental support, and dual careers. Women athletes face difficulties and obstacles in these areas, and they need a safe sports environment free from harassment and abuse (Leiva-Arcas et al., 2021). These difficulties and obstacles faced by female athletes were expressed as reasons for burnout and quitting sports due to physical and psychological pressures (Heidari, 2013). There are differences between genders in sports participation and dropout rates to the disadvantage of women (Leiva-Arcas et al., 2021). Van Tuyckom et al. (2010), in a study on gender inequalities in sports participation covering 25 European countries, state that in European countries, the traditional male dominance in sports is still deeply rooted in most countries, which is in line with the results of this research. In a study by Chukwurah et al. (2022), which investigated gender inequality in the media and the welfare of national teams in line with the Nigerian sports policy, participants stated that there was a wage gap of nearly 80% between male and female national team athletes, and significant inequalities in welfare, and that similar inequalities are also seen in media coverage. Similarly, Jeanes et al. (2021) stated that the images of female soccer players in Australia have no representation in club rooms and on club social media, which in most cases only include images of their male counterparts. Burt (2021) argues that female athletes’ lack of media representation impacts their wages. The lack of media representation of female athletes prevents them from attracting public attention, affecting women’s incomes. Research shows that sports journalism in print media is a man’s world, with more than 90% of articles written by male journalists and more than 85% of stories devoted to male athletes (Katsarova, 2019). These figures indicate women’s lack of presence in sports media.

Regarding the question “What are the participants’ views on good practice examples in the field of gender equality in sport?” the participants’ answers led to the conclusion that role models in their environment are other female athletes and their families, that the visibility of female role models in the media is essential for the elimination of gender stereotypes, and that sport has a transformative power for society. Family, peers, and depictions of sports in popular media can encourage individuals to participate in sports through role modeling (Turman, 2007; Fraser-Thomas et al., 2008). The fact that advertisements treat sports as a masculine field, female athletes who do not exhibit masculine qualities are seen as marginal, and stereotypical portrayals of sports role models may cause sports to be seen as a more privileged field, preventing participation (Rasmussen et al., 2021).

Finally, the study searched for an answer to the question, “What are the participants’ views on strategies/suggestions to support sports activities and gender equality?.” The statements from the participants indicated the need to invest in and promote women’s sports, increase media visibility, make legal and administrative regulations that advocate gender equality in sports, establish policies, ensure cooperation with education and mixed sports projects, and provide support from the immediate environment, as suggestions to support gender equality in sports. These strategies and recommendations are in line with the strategies from the Council of Europe’s Gender in Sport report, which include Women’s participation in sports activities, equal representation, and gender sensitivity in decision-making, gender equality in sports coaching, decreasing gender-based violence in and through sport, and gender stereotypes in sport and the role of the media (EIGE—European Institute for Gender Equality, 2017). Another result from the participants related to this theme is that family and environmental support is an important factor in sports participation and gender equality in sports. Peral-Suárez et al. (2020) stated that there is a relationship between parental support and children’s participation in sports, and that children are significantly more likely to participate in sports, especially when the father takes care of them. However, a radical change in the systemic failures that perpetuate male hegemony can contribute toward equality for future generations (Graham and Blackett, 2022).

The current study contributes to the literature on gender equity in sport by providing insights from different stakeholders and countries, and by offering practical implications for policy makers, practitioners, and researchers. Firstly, existing gender equality policies are not bringing the expected benefits, as the issues most frequently raised by female players were the inequalities and barriers they encounter in this area. Secondly, it is important to listen to the opinion of the female players, as they are the ones who witness these inequalities first-hand, so their opinion is essential to be able to establish effective action policies. Thirdly, isolated sports policies should not be implemented, as it has been observed that the stereotypes present in society are the ones that end up being present in sport, so this issue must be tackled from an integral point of view, addressing all possible sources of inequality. Fourthly, it would be essential to break the glass ceiling for women in sporting participation and in holding important sporting positions, as this conditions their possibilities and aspirations in this area, in which their presence is so important. And fifthly, it is important that female who suffer the consequences of gender stereotypes in the performance of their jobs make this known so that the implementation of gender policies is as targeted and effective as possible. For these reasons, the study also calls for further research on the topic, especially on the physiological and psychological aspects of women’s sport participation.

Considering the above, the present research is not free of limitations. The method used to collect the results was the focus group, by means of a semi-structured interview and spontaneous participation of the participants, what may lead to a shallower approach to the issues. On the other hand, another possible limitation could be the sample included, composed of female athletes at different competitive levels, and a mixed gender-equitable group of teachers, managers, and sports coaches. To achieve a better understanding of the gender perspective in sport, future research could include individual unstructured interviews in order to delve more deeply into the experiences of each of the subjects. Also, the inclusion of different groups with men and women athletes, men and women teachers, coaches, and sport directors, as well as mixed groups, could help reach a better understanding of the topic. However, the present study also has strengths that differentiate it from previous scientific evidence and are important to highlight. Firstly, it is the first study to carry out focus groups with professionals from different sports sectors (female players, coaches, managers, physical education teachers) with the aim of finding out their perception of gender stereotypes present in these areas. Secondly, the aspects considered most relevant by professionals in the different sports sectors are established, which allows proposals to be made for future research and policies aimed at addressing current problems in this area. Thirdly, the most determining factors in which gender stereotypes appear, not only in the field of sports, but also in society, and which can influence sport, have been determined. And fourthly, relevant information has been obtained on the good practices that are currently being carried out in the field of sports for the eradication of gender stereotypes and that are having a relevant impact on the participation of female in the different sports sectors.

## 5 Conclusion

According to the opinion of female athletes and sports managers/teachers/coaches, gender inequality in sport is influenced by the broader social context, where stereotypes, biases and discrimination persist. Furthermore, there are challenges, barriers, and needs that women athletes face in their careers, such as a lack of resources, support, visibility, and recognition. Some strategies and tools to promote gender equity in sport, such as increasing investment, awareness, and education, creating policies and legislation, fostering women’s leadership, and role modeling, and developing mixed and inclusive sports projects are necessary to achieve real gender equality in sport. Thus, gender equity in sports is not only a matter of fairness and justice, but also a matter of social change and empowerment for females. Therefore, it is important to continue to challenge the existing structures and practices that perpetuate gender inequality in sports, and to create more opportunities and spaces for women’s voices and experiences to be heard and valued.

## Data availability statement

The raw data supporting the conclusions of this article will be made available by the authors, without undue reservation.

## Ethics statement

The studies involving humans were approved by Ethics Committee of the Catholic University of Murcia. The studies were conducted in accordance with the local legislation and institutional requirements. Written informed consent for participation in this study was provided by the participants’ legal guardians/next of kin.

## Author contributions

RV-C: Conceptualization, Formal analysis, Funding acquisition, Methodology, Writing – original draft. AM-O: Conceptualization, Formal analysis, Funding acquisition, Methodology, Writing – original draft. YDa: Data curation, Investigation, Resources, Writing – review & editing. AP: Data curation, Methodology, Software, Writing – review & editing. SA: Conceptualization, Formal analysis, Investigation, Supervision, Writing – review & editing. LM: Conceptualization, Formal analysis, Funding acquisition, Methodology, Writing – original draft. NG-G: Conceptualization, Formal analysis, Funding acquisition, Methodology, Writing – original draft. ÖB: Data curation, Investigation, Resources, Writing – review & editing. FM: Data curation, Investigation, Methodology, Software, Writing – review & editing. OM: Conceptualization, Formal analysis, Investigation, Supervision, Writing – review & editing. LA-C: Conceptualization, Formal analysis, Funding acquisition, Methodology, Writing – original draft. AL-A: Conceptualization, Formal analysis, Funding acquisition, Methodology, Writing – original draft. YDo: Data curation, Investigation, Resources, Writing – review & editing. AF: Data curation, Investigation, Methodology, Software, Writing – review & editing. CP-R: Conceptualization, Formal analysis, Funding acquisition, Methodology, Writing – original draft. FE-R: Conceptualization, Formal analysis, Funding acquisition, Methodology, Writing – original draft. MA-S: Conceptualization, Formal analysis, Funding acquisition, Methodology, Writing – original draft.
